# Pathogenic variants in human DNA damage repair genes mostly arose in recent human history

**DOI:** 10.1186/s12885-024-12160-6

**Published:** 2024-04-04

**Authors:** Bojin Zhao, Jiaheng Li, Siddharth Sinha, Zixin Qin, Si Hoi Kou, Fengxia Xiao, Huijun Lei, Tianhui Chen, Wenming Cao, Xiaofan Ding, San Ming Wang

**Affiliations:** 1 Cancer Centre and Institute of Translational Medicine, Faculty of Health Sciences, University of Macau, Taipa, 999078 Macau SAR China; 2 Department of Cancer Prevention, Zhejiang Cancer Hospital, Hangzhou, 310022 China; 3 Department of Breast Medical Oncology, Zhejiang Cancer Hospital, Hangzhou, 310022 China; 4 Hangzhou Institute of Medicine, Chinese Academy of Sciences, Hangzhou, 310018 China

**Keywords:** DNA damage repair, Pathogenic variants, Evolutionary origin, Phylogenetic, Archaeological

## Abstract

**Background:**

Genome stability is maintained by the DNA damage repair (DDR) system composed of multiple DNA repair pathways of hundreds of genes. Germline pathogenic variation (PV) in DDR genes damages function of the affected DDR genes, leading to genome instability and high risk of diseases, in particular, cancer. Knowing evolutionary origin of the PVs in human DDR genes is essential to understand the etiology of human diseases. However, answer to the issue remains largely elusive. In this study, we analyzed evolutionary origin for the PVs in human DDR genes.

**Methods:**

We identified 169 DDR genes by referring to various databases and identified PVs in the DDR genes of modern humans from ClinVar database. We performed a phylogenetic analysis to analyze the conservation of human DDR PVs in 100 vertebrates through cross-species genomic data comparison using the phyloFit program of the PHAST package and visualized the results using the GraphPad Prism software and the ggplot module. We identified DDR PVs from over 5000 ancient humans developed a database to host the DDR PVs (https://genemutation.fhs.um.edu.mo/dbDDR-AncientHumans). Using the PV data, we performed a molecular archeological analysis to compare the DDR PVs between modern humans and ancient humans. We analyzed evolution selection of DDR genes across 20 vertebrates using the CodeML in PAML for phylogenetic analysis.

**Results:**

Our phylogenic analysis ruled out cross-species conservation as the origin of human DDR PVs. Our archeological approach identified rich DDR PVs shared between modern and ancient humans, which were mostly dated within the last 5000 years. We also observed similar pattern of quantitative PV distribution between modern and ancient humans. We further detected a set of *ATM*, *BRCA2* and *CHEK2* PVs shared between human and Neanderthals.

**Conclusions:**

Our study reveals that human DDR PVs mostly arose in recent human history. We propose that human high cancer risk caused by DDR PVs can be a by-product of human evolution.

**Supplementary Information:**

The online version contains supplementary material available at 10.1186/s12885-024-12160-6.

## Background

Human genome is constantly attacked by various internal and external assaults. The damaged DNA must be timely repaired to maintain genome stability and prevent pathogenic consequences. During the evolutionary process, life system has developed an efficient DNA damage repair (DDR) machinery consisting of multiple pathways and hundreds of genes. Each pathway is responsible for repairing one or more specific types of DNA damage [1]. For example, the homologous recombination pathway consisting of BRCA1, BRCA2, RAD51, PALB2 etc., repairs double strand DNA break. However, DDR genes are prone to germline variation. While many variants can be beneficial or neutral, a portion of the variants is pathogenic (deleterious in biological term) as they damage function of the affected DDR genes, letting the damaged DNA unrepaired, resulting in genome instability [2]. The pathogenic variants (PVs) in DDR genes are well determined as the genetic predisposition for high risk of diseases, particularly cancer [3, 4]. Based on the “Two-Hit” theory, the DDR PVs can function as the first hit in initiating the oncogenic process [5], as exampled by *BRCA1* PVs that damage the double-strand DNA break repairing function of homologous recombination pathway leading to high risk of breast cancer [6–8]. Furthermore, DDR PVs are widely used as specific diagnostic markers for cancer risk prediction, e.g., *BRCA1* PVs are often used as the markers to predict breast cancer risk [9] and guide cancer treatment, e.g., as the indicator for using PARP inhibitors (PARPi) to treat breast cancer with *BRCA1* PVs [10]. In addition, it has been observed that human has much higher cancer risk than many other animals such as elephant, whales, and even the closest human relative chimpanzee [11–13], which may be attributed by the human-specific DDR PVs.

To understand the roles of DDR PVs as the genetic predisposition to human diseases, it is essential to know the evolutionary origin of the DDR PVs in the humans. Despite decades’ efforts made in studying the relationship between human DDR PVs and human diseases, however, our knowledge for this fundamental issue remains very limited [14–18]. So far, evolutionary study has been performed in a small number of DDR variants in a few DDR genes, of which most were variant of uncertain significance (VUS) and benign variants (BVs), but few were pathogenic variants, or were the genetic loci without actual variants [19]. Therefore, the results mainly reflect the evolutionary conservation of genetic polymorphism in DDR genes (Table 1, Supplementary Table 1). Lack of the knowledge for the evolutionary origin of human DDR PVs prevents our deeper understanding for the roles of genetic predisposition in human diseases, particularly the etiology of human cancer.

**Table 1:** Summay of previous evolutionary studies in DDR gene variation

Class (ClinVar)	Number of variants in the affected DDR genes						Total (%)
	*BRCA1*	*BRCA2*	*XPC*	*XPA*	*RAD51*	*POLD1*	
Pathogenic	4	1	9	3	1	0	18 (14.2)
Benign	36	8	0	0	0	0	44 (34.6)
Uncertain significance	45	2	0	0	0	1	48 (37.8)
Conflicting interpretations	12	2	0	0	1	0	15 (11.8)
Not provided	1	1	0	0	0	0	2 (1.6)
Total (%)	98 (77.2)	14 (11.0)	9 (7.1)	3 (2.4)	2 (1.6)	1 (0.8)	127 (100)

We considered that only three possible origins exist for human DDR PVs: 1) from the common ancestry between human and non-human species through cross-species conservation; 2) from human itself during the human evolution process; and 3) from both sources. In our previous study, we observed that the PVs in human DDR genes of *BRCA1* and *BRCA2*, *TP53*, *MUTHY* and *PALB2* were not inherited from cross-species conservation but originated during recent human evolutionary history [20–23]. The same pattern of PV origin in these DDR genes seems suggesting that the 2nd possibility could be the origin for the PVs in all human DDR genes that they were originated from human itself during human evolution process. To test this possibility, we expanded the study by applying the phylogenetic and archaeological approaches used in these DDR genes to characterize the PVs in 169 human DDR genes. In the phylogenetic analysis of searching the DDR PVs of modern humans in 100 non-human vertebrates across 8 clades, we were unable to find evidence to support evolution conservation as the origin; in the archaeological analysis of comparing the DDR PVs of modern humans to the DDR PVs from over 5000 ancient humans, we observed rich sharing of modern human DDR PVs in the ancient humans dated mostly within the last 5000 years. Our study reveals that the PVs in human DDR genes mostly arose during recent human history, and highlights the possibility that the high cancer risk of modern humans caused by DDR PVs can be a by-product of human evolution.

## Methods

### Sources of DDR genes and PVs

The 169 DDR genes were identified by referring to the “Replication and repair” of KEGG [24] (https://www.genome.jp/kegg/pathway.html#cellular) and Human DNA Repair Genes [10] (https://www.mdanderson.org/documents/Labs/Wood-Laboratory/human-dna-repair-genes.html). In using genetic variation for clinical applications, a genetic variant is usually classified into one of the classes of pathogenic, likely pathogenic, uncertain significance, likely benign, or benign. Pathogenic and likely pathogenic variants (PVs) are directly relevant to clinical applications for disease diagnosis, treatment and prognosis, and were the focus of our current study. The DDR PVs were extracted from ClinVar Database (https://www.ncbi.nlm.nih.gov/clinvar/, build 20,221,119), and used in the study to represent the DDR PVs in modern humans. All ClinVar PVs used were with review status of over three stars, and these with conflicting classifications were excluded in the study.

### Sources of genomic sequence data of 100 vertebrates and ancient humans

The reference genome sequences for the 100 vertebrate species in 8 clades were from UCSC Genome Browser [25]. The genomic sequences of 5031 ancient humans dated between 45,045 and 100 years BP (before present), Neanderthals dated between 80,000 years BP and 48,000 years BP and Denisovans dated 78,000 years BP were from the European Nucleotide Archive (https://www.ebi.ac.uk/ena/) and the server of Max Planck Institute for Evolutionary Anthropology (http://cdna.eva.mpg.de/neandertal/, http://cdna.eva.mpg.de/denisova/).

### Phylogenetic genome mapping analysis

**Fig. 1:**
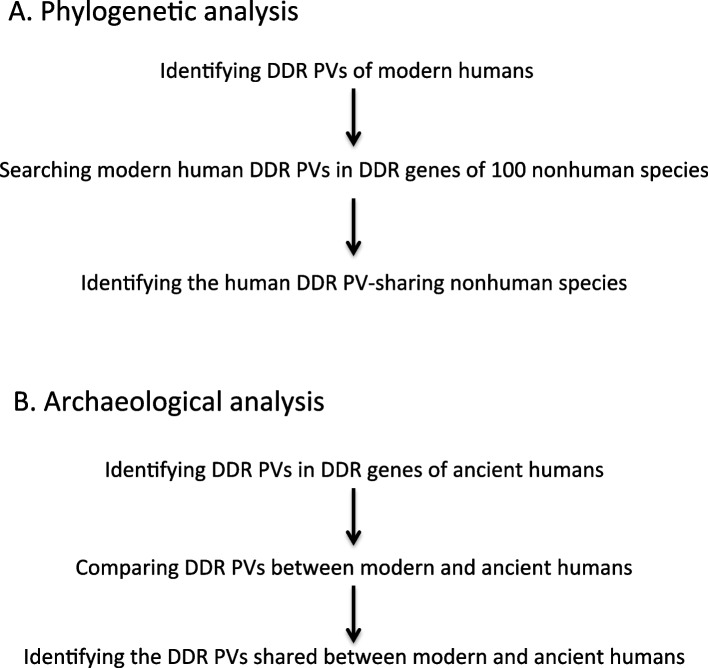
Outline of study design. The study included two major parts: Phylogenetic analysis and archeological analysis, with the aim to determine the origin of DDR PVs in modern humans

DDR PVs in modern humans were searched in 100 vertebrate species following the procedures [20] (Fig. 1). Briefly, sequence alignment was processed through “Multiz Alignments of 100 Vertebrates” through UCSC Genome Browser. The phyloFit program in the PHAST package was applied to build up the phylogenetic tree model for the 100 vertebrate species [26]. Multiple sequence alignment across species was performed by using Multyz and Lastz programs [27, 28]. The tuning of the scoring matrix, parameters for pairwise alignment, and chaining for each species were based on the phylogenetic distance from the references. High-scoring chains were directly placed along the genomes and the remaining gaps were filled with lower-scoring chains to create alignment nets. A Python-based Selenium module (https://www.selenium.dev/) was used to collect the aligned data from “Multiz Alignments of 100 Vertebrates” with output as Excel tables. Figures were generated using GraphPad Prism (version 9.4.2 for Windows, GraphPad Software).

### Archeological comparison of DDR PVs between modern and ancient humans

The process followed the procedures [20]. Briefly, BAM files were aligned to the human reference genome GRCh37 (hg19). DNA sequences of the DDR genes were extracted from the aligned sequences using the view command of SAMtools. The position of each gene was referred to GeneCards (https://www.genecards.org/). The sequences for each DDR gene were checked using mapDamage 2.0 [29]. For the variants in ancient humans, a base-recalibrating Perl script was used to remove the false variants caused by deamination in ancient DNA (aDNA) [30]. Variant calling was performed using the mpileup commands of BCFtools [31] with the minimum base quality set as one. The called variants were annotated using ANNOVAR [32], with the references of ClinVar, NCBI RefGene database, dbSNP build 155 and the 1000 Genomes. DDR PVs from ClinVar were searched in ancient humans, Neanderthals and Denisovans. Python scripts were applied to annotate the variants including carrier accession number, dated time and location of ancient samples, sequence depth, cDNA reference sequences, protein reference sequences, HGVS cDNA and protein description. GraphPad Prism was used to compare the DDR PVs between ancient and modern humans, R scripts were used to visualize the annotated data and figures. Python scripts based on the Pandas module (https://pandas.pydata.org/) were used to compare the DDR PVs between ancient and modern humans. GraphPad Prism was used to visualize the annotated data.

### Evolution selection for DDR genes

The 73 DDR genes with the PVs shared between modern and ancient humans were used for the study. The analyses were performed in 20 vertebrate species of chimpanzee, gorilla, orangutan, marmoset, mouse, rat, guineapig, rabbit, cow, pig, horse, dog, elephant, opossum, platypus, chicken, zebra finch, frog, fugu, and zebrafish. The coding DNA and protein reference sequences for the 73 DDR genes in each species were retrieved from NCBI RefSeq database (https://www.ncbi.nlm.nih.gov/refseq/, accessed January 17, 2023). The pairwise sequence alignment was performed using the ClustalW algorithm [33, 34], and multiple sequence alignment was performed using Molecular Evolutionary Genetics Analysis (MEGA) version 11 [35]. GBLOCKS program was used to filter the alignments to eliminate potential false positive results [36]. Phylogenetic trees were built using the maximum likelihood under the Tamura-Nei model with the minimum of 1000 bootstrap replicas. To test evolution selection, the maximum likelihood-based CODEML in PAML version 4.9 [37] was used to calculate the dN/dS ratio (ω) between nonsynonymous and synonymous substitution. ω > 1 was defined as positive selection, ω < 1 as negative selection, and ω = 1 as neutral. CODEML program model 2 was used to estimate the substitution ratio for the species branches, with the run-mode set to − 2 for the pairwise comparison of the substitution ratio between species.

### Database construction

An open-access database “dbDDR-Ancient humans” was constructed to host the DDR PVs in ancient humans shared with modern humans (https://genemutation.fhs.um.edu.mo/dbDDR-AncientHumans). Briefly, HTML, CSS and JavaScript were used to design the front-end of the database. HTML laid out the content and structure, CSS managed the typography design features, and JavaScript created front-end communication. The database back-end was implemented with the LAMP stack, a bundle of open-source software technologies including the operation system Linux (CentOS 7, https://www.centos.org/), the web server Apache (Apache 2.4.52, https://httpd.apache.org/), the database server MySQL (5.6.50, https://www.mysql.com/), and the scripting language PHP (PHP 7.3, https://www.php.net/).

### Statistical analysis

The Pearson correlation coefficient (r) was used to calculate the linear correlation of DDR PV abundance between ancient humans and modern humans, and a two-tailed hypothesis test was used to test the significance of linear correlation. Kruskal–Wallis one-way analysis was used to compare the DDR PVs shared between different clades.

## Results

### Human DDR PVs in non-human vertebrates

**Fig. 2:**
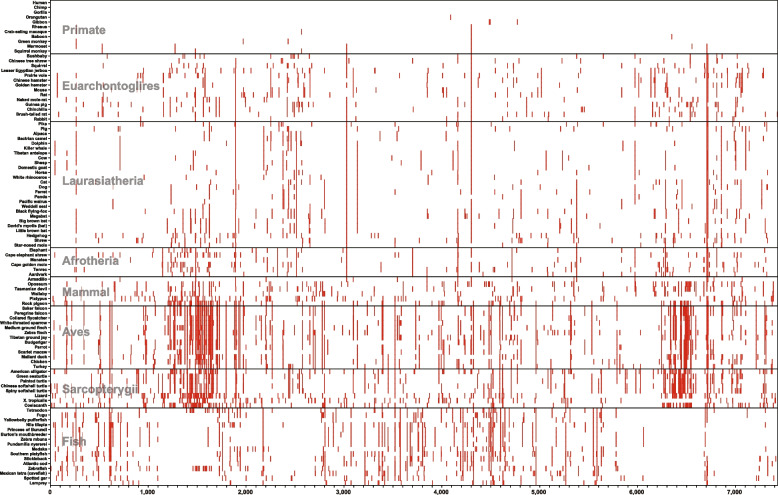
Phylogenetic analysis of human DDR PVs in 100 vertebrate species. The 7432 single nucleotide PVs in 87 DDR genes from ClinVar database were searched in 100 vertebrate species with 8 clades of Primate, Euarchontoglires, Laurasiatheria, Afrotheria, Mammalia, Aves, Sarcopterygii and Fish. The results showed that the majority of human DDR PVs were not shared, whereas 1497 (20.14%) were shared with non-human vertebrate species mostly distal to human such as the species within Aves and Sarcopterygii, and rarely with species within Primate. Y-axis: species; X-axis: PVs in DDR genes. Red: human PVs shared with other species

The ClinVar database contained 20,272 PVs in 91 of the 169 DDR genes, of which 7432 were single nucleotide PVs, representing the DDR PVs currently known in modern humans (https://www.ncbi.nlm.nih.gov/clinvar/, build 20,221,119). Using the 7432 single nucleotide PVs, we performed a phylogenetic analysis to investigate whether the DDR PVs in modern humans could be originated from cross-species conservation. We searched the human DDR PVs in 100 vertebrate species distributed in 8 clades of Primate, Euarchontoglires, Laurasiatheria, Afrotheria, Mammalia, Aves, Sarcopterygii and Fish. We identified 1497 (20.14%) of the 7432 human DDR PVs shared in 97 (98.0%) of the 100 non-human vertebrate species. However, the species sharing with human DDR PVs didn’t follow the order of evolutionary tree but were mostly distal from the humans, particularly in the clades of Aves and Sarcopterygii (Fig. 2, Supplementary Table 2). Although there were 8 species in Primate sharing 53 human DDR PVs, Chimpanzee as the closest relatives to human and Gorilla didn’t share any human DDR PVs; Orangutan with divergent time of 15.2 million years ago (MYA) from human shared only one human DDR PV; whereas Bushbaby, the most distal to human with divergent time of 74 MYA, shared 23 human DDR PVs (Table 2) [38]. The results from phylogenetic analysis didn’t support cross-species conservation as the origin for nearly all DDR PVs in modern humans, if there could be any.

**Table 2:** Species in the clade of Primate sharing human DDR PVs

Species (MYA^a^)	Gene	Shared human DDR PVs	
		cDNA	Protein
Orangutan (15.2)	*PMS2*	c.803 + 1G > A	p.(Tyr268^*^)
Gibbon (19.5)	*TP53*	c.537 T > A	p.(His179Gln)
	*TP53*	c.489C > A	p.(Tyr163^*^)
	*DNA2*	c.593G > A	p.(Arg198His)
Rhesus (28.8)	*ATM*	c.3994-159A > G	p.(=)
	*TP53*	c.887A > G	p.(His296Arg)
Crab-eating macaque (28.8)	*ATM*	c.3994-159A > G	p.(=)
	*FANCA*	c.14G > A	p.(Trp5^*^)
	*TP53*	c.887A > G	p.(His296Arg)
Baboon (28.8)	*TP53*	c.887A > G	p.(His296Arg)
	*BRCA1*	c.3122C > G	p.(Ser1041^*^)
Green monkey (28.8)	*ATM*	c.3994-159A > G	p.(=)
	*BRCA2*	c.9501G > A	p.(=)
	*FANCA*	c.2982-192A > G	p.(=)
	*TP53*	c.887A > G	p.(His296Arg)
Marmoset (38)	*ATM*	c.3994-159A > G	p.(=)
	*ATM*	c.2341C > T	p.(Gln781^*^)
	*BRCA2*	c.1642C > T	p.(Gln548^*^)
	*MLH1*	c.208-3C > T	p.(=)
	*TP53*	c.887A > G	p.(His296Arg)
	*BRCA1*	c.850C > T	p.(Gln284^*^)
	*POLK*	c.1284G > A	p.(=)
Squirrel monkey (38)	*ATM*	c.2341C > T	p.(Gln781^*^)
	*BRCA2*	c.1642C > T	p.(Gln548^*^)
	*BRCA2*	c.4689G > A	p.(Trp1563^*^)
	*FANCA*	c.14G > A	p.(Trp5^*^)
	*MLH1*	c.208-3C > T	p.(=)
	*TP53*	c.887A > G	p.(His296Arg)
	*POLK*	c.1284G > A	p.(=)
Bushbaby (74)	*ATM*	c.3382C > T	p.(Gln1128^*^)
	*BRCA2*	c.2651C > A	p.(Ser884^*^)
	*BRCA2*	c.2978G > A	p.(Trp993^*^)
	*BRCA2*	c.4689G > A	p.(Trp1563^*^)
	*BRCA2*	c.5263G > T	p.(Glu1755^*^)
	*BRCA2*	c.5791C > T	p.(Gln1931^*^)
	*FANCA*	c.3765 + 2C > T	p.(=)
	*FANCA*	c.2504 + 134A > G	p.(=)
	*FANCA*	c.2021C > A	p.(Ser674^*^)
	*FANCA*	c.1567-20A > G	p.(=)
	*FANCA*	c.683C > G	p.(Ala228Gly)
	*FANCC*	c.1490G > A	p.(Trp497^*^)
	*MLH1*	c.208-3C > T	p.(=)
	*PMS2*	c.1606C > T	p.(Gln536^*^)
	*TP53*	c.887A > G	p.(His296Arg)
	*FANCG*	c.346C > T	p.(Gln116^*^)
	*POLH*	c.2074A > G	p.(Thr692Ala)
	*BRCA1*	c.4185 + 4105C > T	p.(=)
	*BRCA1*	c.1058G > A	p.(Trp353^*^)
	*POLK*	c.1284G > A	p.(=)
	*POLK*	c.2033C > T	p.(Ser678Phe)
	*RNASEH2B*	c.58G > C	p.(Val20Leu)
	*PALB2*	c.1451 T > A	p.(Leu484^*^)
	*PALB2*	c.1675C > T	p.(Gln559^*^)

^a^MYA (million year ago) from human based on TIMETREE5 (timetree.org)

^*^MYA (million year ago) refers to the time of the species from human based on TIMETREE5 (timetree.org)

### Human DDR PVs in ancient humans

We then performed an archaeological analysis to know whether the DDR PVs in modern humans could arise from the evolution process of human itself. We collected the genome sequences from publicly available 5031 ancient human individuals dated between 45,045 and 100 years BP (Supplementary Table 3), and called the DDR PVs from the ancient humans. Comparison between the 7432 DDR PVs in 87 DDR genes of modern humans and the PVs of the 5031 ancient human individuals identified 1266 (5.3%) DDR PVs with 1019 ancient carriers in 73 (84.0%) of the 87 DDR genes. We developed a database to host the DDR PVs identified in ancient humans (Table 3, Table 4, Supplementary Table 4, https://genemutation.fhs.um.edu.mo/dbDDR-AncientHumans).

**Table 3:** DDR PVs identified in ancient humans. Summary

DDR pathways	DDR genes	Ancient humans		Modern humans^b^	
		Gene with PVs (%)	No. PVs	Genes with PVs (%)	No. PVs
Fanconi anemia (FA)	49	27 (55)	615	30 (61)	926
Homologous Recombination (HR)	37	21 (57)	636	21 (57)	916
Mismatch Repair (MMR)	22	8 (36)	225	8 (36)	188
Nucleotide Excision Repair (NER)	42	13 (31)	95	13 (31)	163
Nonhomologous end joining (NHEJ)	13	6 (46)	64	7 (54)	129
DNA damage response (DDR)	15	3 (20)	82	5 (33)	86
Base Excision Repair (BER)	32	7 (22)	46	7 (22)	72
DNA replication (DR)	34	12 (35)	36	11 (32)	36
Total^a^	169	73 (43)	1266	81 (48)	1781

^a^ Nonredundant numbers

^b^ Referred from Qin et al., 2022 [39]

**Table 4:** DDR PVs identified in ancient humans. Number of DDR PVs identified in ancient humans

Gene	PVs	Gene	PVs
*ATM*	147	*RAD51*	5
*BRCA2*	122	*XPA*	5
*BRCA1*	116	*ATR*	4
*FANCA*	67	*DCLRE1C*	4
*MLH1*	66	*ERCC4*	4
*MSH2*	59	*LIG4*	4
*TP53*	50	*NHEJ1*	4
*PALB2*	48	*RAD54L*	4
*MSH6*	46	*RNASEH2C*	4
*RAD50*	38	*SLX4*	4
*MUTYH*	33	*SSBP1*	4
*BRIP1*	33	*XRCC4*	4
*PMS2*	32	*ERCC1*	3
*ERCC6*	30	*FAN1*	3
*BARD1*	28	*RNASEH1*	3
*CHEK2*	28	*TELO2*	3
*NBN*	24	*FANCF*	2
*BLM*	21	*GTF2H5*	2
*FANCC*	17	*LIG1*	2
*RAD51D*	16	*LIG3*	2
*MSH3*	15	*MCM2*	2
*FANCD2*	12	*RBBP8*	2
*RAD51C*	12	*XRCC2*	2
*XPC*	12	*MCM7*	2
*ERCC2*	11	*RAD51B*	1
*FANCI*	11	*DDB2*	1
*FANCG*	10	*DNA2*	1
*MRE11*	10	*FANCL*	1
*ERCC3*	9	*MCM5*	1
*ERCC8*	8	*POLD1*	1
*RNASEH2B*	8	*TOP3A*	1
*ERCC5*	7	*UBE2T*	1
*RNASEH2A*	7	*UIMC1*	1
*FANCE*	6	*UNG*	1
*FANCM*	6	*XRCC1*	1
*NTHL1*	6	*MCM4*	1
*POLH*	5		

Of the 1019 ancient carriers, 959 had dated time, of which 717 (74.8%) were within 5000 years BP, 214 (22.3%) between 5000 and 10,000 years BP, and 28 (2.9%) before 10,000 years BP. The carriers of *ATM* c.3077 + 1G > A was the oldest dated 37,470 years BP in Shamanka II, Russia and 34,425 years BP in Salkhit Valley, Mongolia; The carriers of *MSH2* c.1204C > T p.(Gln402*), *MUTYH* c.437G > A p.(Trp146*) and *RNASEH2A* c.69G > A p.(=) were the youngest dated 190 years BP in Vanuatu and Germany (Supplementary Table 4). A total of 67 PVs (5.3% of the 1266 DDR PVs) in 33 DDR genes (45.2% of the 73 DDR genes) were recurrent in ancient carriers dated within the last 10,000 years.

**Fig. 3:**
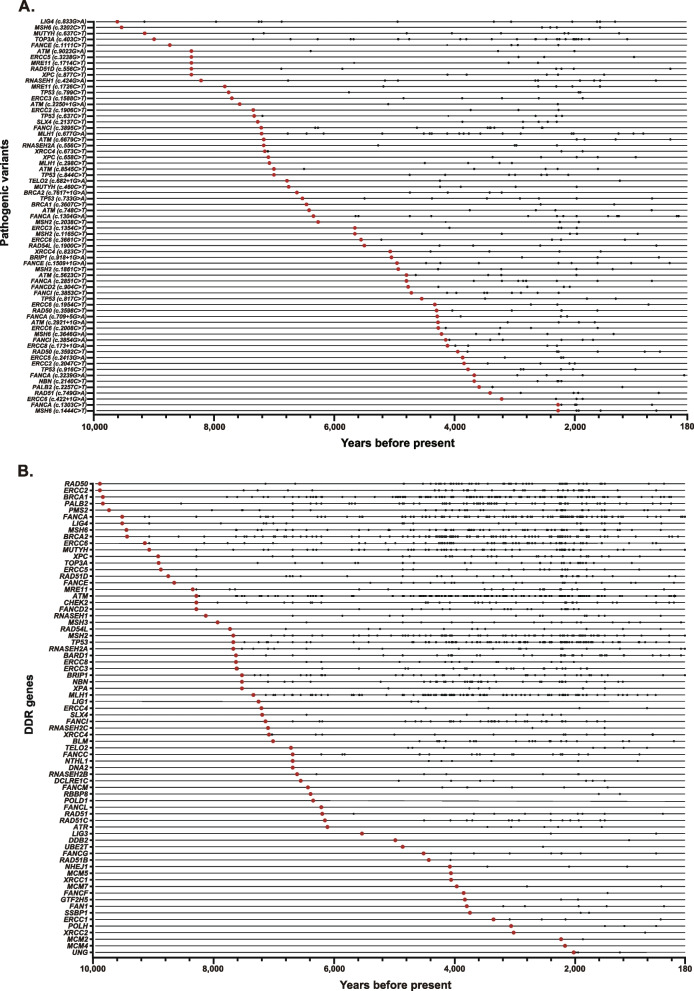
Timing of DDR PVs of ancient humans shared with modern humans. **A** Timing distribution of 67 recurrent PVs in 33 DDR genes shared between modern and ancient humans within 10,000 years BP (a few longer than 10,000 years BP were not included due to space limitation). The larger red dot in each PV line represents the earliest identified PV carrier, and the smaller black dot (s) in the same line correspond to the later carriers of the same PV. The line to the left of the larger red dot indicates the presence of PV-free sample(s) older than the first PV carrier, showing that older ancient sample(s) were present for all first identified recurrent PVs carriers, Supplementary Table 7), the line to the right of the last smaller black dot denotes the presence of PV-free sample(s) younger than the last PV carriers. **B** Different PVs in 73 DDR genes shared between modern and ancient humans within the last 10,000 years (a few longer than 10,000 years BP were not included due to space limitation). Each dot represents a single PV. The larger red dot in each DDR gene line represents the earliest PV identified in the gene, and the smaller black dot (s) in the same line represents different PVs detected later in the same DDR gene. Most of the PVs were present in the carriers dated within the last 5000 year BP. It shows that older ancient samples were present for all firstly identified PVs carriers (Supplementary Table 7)

Of the 67 recurrent PVs in ancient carriers shared with modern humans*, LIG4* c.833G > A was the earliest PV identified in a Brazilian carrier dated 9550 years BP, and *MSH6* c.1407 T > A was the youngest PV identified in a Russian carrier dated 1070 years BP (Fig. 3A); Of the 73 DDR genes with PVs shared with modern humans, *RAD50* c.3G > A was the earliest PV, identified in a Chinese carrier dated 9922 years BP; and *UNG* c.685C > T was the youngest PV identified in a Mongolian carrier dated 2040 years BP (Fig. 3B); of the DDR pathways affected by the PVs, homologous recombination (HR) pathway had the highest PV number of 636 in 21 (67.7%) of the 37 genes in HR pathway, Fanconi anemia (FP) pathway had the 2nd highest PV number of 615 in 27 (55.1%) of the 49 genes in FP pathway (Table 3). We also identified 5 DDR PVs in 5 Neanderthals dated between 80,000 and 50,000 years BP including *ATM* c.5918 + 1G > A p.(=), c.8584 + 1G > A p.(=) and c.7089 + 1G > A p. (=), *BRCA2* c.9076C > T p.(Gln3026*), and *CHEK2* c.988C > T p.(Gln330*) (Supplementary Table 5).

### Comparison of DDR PV content and abundance between ancient and modern humans

In our previous study, we identified 1781 DDR PVs in 16 global ethnic human populations [39]. Using the 1781 DDR PVs to represent the DDR PV in modern humans and the 1266 DDR PVs to represent the DDR PV in ancient humans, we compared the DDR PV content between the modern and ancient humans. The results showed two different patterns (Table 3): 1) the number of DDR genes carrying PVs didn’t change much, but the total number of PVs increased substantially from ancient humans to modern humans. For example, 21 genes in the homologous recombination pathway were affected by PVs in both ancient and modern humans, but the number of PVs in modern humans was much higher than in ancient humans (916 to 636). Similarly, with the same number of 13 PV-affected genes in nucleotide excision repair pathway, the number of PVs increased from 95 in ancient humans to 163 in modern humans; 2) the numbers of PV-affected genes and PVs remained stable between ancient and modern humans. This was present in mismatch repair pathway, DNA damage response pathway and DNA replication pathway. The results suggest that DDR PVs in different DDR pathways were under differential selection during recent human evolutionary process.

**Fig. 4:**
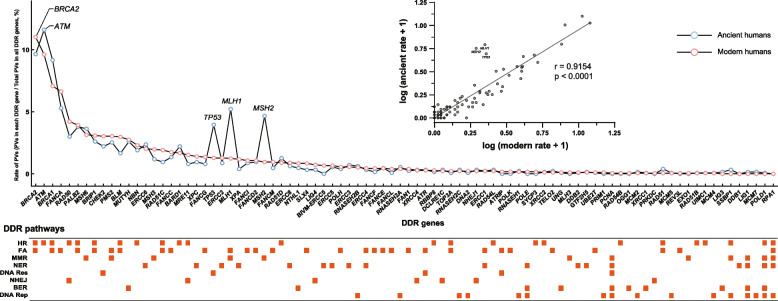
Comparison of DDR PV abundance between ancient humans and modern humans. The comparison was made between the 1781 DDR PVs identified in global modern humans [39] and the 1266 DDR PVs identified in ancient humans in this study. The rate of PVs in each DDR gene in modern or ancient humans was calculated as the number of PVs in each gene / total number of PVs in all DDR genes. Each rectangle in the lower part refers to the PV-containing DDR genes in the affected DDR pathway. It shows that DDR PVs between ancient and modern humans shared similar prevalent distribution, except the PVs of *TP53*, *MLH1* and *MSH2* at higher abundance in ancient humans than in modern humans

We further compared the abundance of individual PVs between modern humans and ancient humans by using the ratio of individual DDR PV / total DDR PVs. We observed similar pattern for most DDR PVs between modern humans and ancient humans (Fig. 4): *ATM*, *BRCA2*, and *BRCA1* PVs had the highest abundance among all DDR genes (Table 4, Fig. 4). ATM has auto-phosphorylation kinase activity in phosphorylating its serine residues of Ser 367, Ser 1893, and Ser 1981, but none of the 147 shared PVs were located at these sites suggesting that these positions didn’t tolerate changes (Supplementary Table 4); CHEK2 is a serine/threonine-protein kinase in regulating cell cycle and apoptosis, repairing double-strand DNA damage through the ATM-CHK2-p53 pathway, and *CHEK2* PVs increase breast cancer risk [40, 41]. There were 28 *CHEK2* PVs with 42 carriers dated within 10,000 years BP (Table 5). Among these PVs, *CHEK2* c.283C > T p. (Arg95*) was the most common one, with 4 carriers in Turkey (time unknown), Russia (3435 years BP), Czech (time unknown), and China (2160 years BP), although it had low frequency in modern human (rs587781269, allele frequency of 0.000007961 in gnomAD). The popular PV *CHEK2* 1100del in modern humans was not within the 28 *CHEK2* PVs, implying its lower abundance in ancient humans. As the exceptions, PVs in *TP53*, *MLH1* and *MSH2* had much higher abundance in ancient human than in modern humans (*TP53*: 4.0% in ancient humans versus 1.3% in modern humans; *MLH1*: 5.2% in ancient humans versus 1.2% in modern humans; *MSH2*: 4.7% in ancient humans versus 1.0% in modern humans), suggesting that they were suppressed during human evolutionary process.

**Table 5:** *CHEK2* pathogenic variants identified in ancient humans

Time (BP)	Variants		dbSNP155	Position	Variation type	Ancient carriers	
	HGVSc	HGVSp				Location	Number
32,823	c.409C > T	p.(Arg137*)	rs730881701	exonic	stopgain	Russia, Italy	2
10,000	c.1375 + 1G > A	p.(=)	rs759706360	splicing	–	Brazil	1
8315	c.433C > T	p.(Arg145Trp)	rs137853007	exonic	nonsynonymous	Turkey, Sudan, China	3
7963	c.31C > T	p.(Gln11*)	rs1349961118	exonic	stopgain	Ukrayina, Mongolia	2
7480	c.291G > A	p.(Trp97*)	rs2054312626	exonic	stopgain	Finland, Mongolia	2
6856	c.1297C > T	p.(Gln433*)	rs1555913494	exonic	stopgain	Russia	1
6713	c.319 + 1G > A	p.(=)	rs765080766	splicing	–	Russia, Ukraine	2
6415	c.205C > T	p.(Gln69*)	rs768384031	exonic	stopgain	Chile	1
6386	c.625C > T	p.(Gln209*)	rs1569149953	exonic	stopgain	Russia, Croatia	2
6238	c.683 + 1G > A	p.(=)	rs786203650	splicing	–	Russia	1
4503	c.1315C > T	p.(Gln439*)	rs778989252	exonic	stopgain	Russia	1
4498	c.100C > T	p.(Gln34*)	rs1231012263	exonic	stopgain	China	1
4462	c.1555C > T	p.(Arg519*)	rs200432447	exonic	stopgain	Mongolia, China	2
4272	c.279G > A	p.(Trp93*)	rs587782070	exonic	stopgain	Russia	1
3681	c.1461 + 2 T > C	p.(=)	rs779844113	splicing	–	Denmark	1
3626	c.232C > T	p.(Gln78*)	rs1555932341	exonic	stopgain	Russia	1
3556	c.151C > T	p.(Gln51*)	rs587781592	exonic	stopgain	Sweden	1
3435	c.283C > T	p.(Arg95*)	rs587781269	exonic	stopgain	Turkey, Russia, Czech, China	4
3328	c.1095 + 1G > A	p.(=)	rs768172525	splicing	–	Turkey, Russia	2
3000	c.908 + 2 T > C	p.(=)	rs1601752066	splicing	–	Russia	1
2064	c.592 + 1G > A	p.(=)	rs1601822722	splicing	–	Turkey, Russia	2
1988	c.1528C > T	p.(Gln510*)	rs886039512	exonic	stopgain	Russia	1
1646	c.85C > T	p.(Gln29*)	rs761494650	exonic	stopgain	Turkey, Russia	2
450	c.341G > A	p.(Trp114*)	rs1555927374	exonic	stopgain	Turkey	1
372	c.1050del	p.(Glu351Argfs*14)	rs2052610542	exonic	deletion	Italy	1
NA	c.79C > T	p.(Gln27*)	rs376736188	exonic	stopgain	Turkey	1
NA	c.1009-1G > A	p.(=)	rs1555914382	splicing	–	Turkey	1
NA	c.1486C > T	p.(Gln496*)	rs756250205	exonic	stopgain	Turkey	1

### Arisen time of founder DDR PVs in ancient humans

Many DDR PVs have been determined as founder PVs for cancer in different human ethnic populations and their arisen times have been determined by haplotyping analysis. We searched the literature to identify the haplotyping-determined founder DDR PVs shared between ancient and modern humans. In total, we identified 68 founder DDR PVs dated from 8675 to 180 years BP in 7 DDR genes of *BRCA1, BRCA2, MLH1, MSH2, MSH6, MUTYH* and *TP53*, of which 44 PVs (64.7%) were in *BRCA1* (Table 6). In chronological order, the three *MUTYH* PVs of c.1103G > A p.Gly368Asp, c.452A > G p.Tyr151Cys, and c.849 + 3A > C were dated 8675 years BP, 7500 years BP, and 2075 years BP, respectively [42, 43]; a group of 21 *BRCA1* PVs in Pakistani were dated 3800 year BP [44]; of the 14 *BRCA2* PVs, the oldest one was the *BRCA2* c.9026_9030del in Spaniard dated 2760 years BP [45]; the *TP53* c.1010G > A in Brazilian was arisen 2000 years BP [46]; the *BRCA1* c.68_69del and c.5266dup, and *BRCA2* c.5946del in Ashkenazi Jewish were arisen 1500–750, 1800, and 580 years BP, accordingly [47]; the *BRCA1* c.4035delA in Balts was arisen 1550 years BP [48], the *BRCA2* c.771_775del in Icelandic population was arisen 500 years BP [49], the *BRCA2* c.7480C > T and *BRCA2* c.8327 T > G in Finnish was arisen 400–200 BP, and 220–140 years BP, accordingly [50]. Except *MUTYH* c.1103G > A and c.452A > G, all founder DDR PVs identified so far were dated within the past 4000 years BP.

**Table 6:** DDR founder PVs and their arisen time

Gene	cDNA	Protein	Pubmed ID	Ethnic population	Year BP
*BRCA1*	c.66dup	p.Glu23Argfs*18	35377490	Pakistani	3800
*BRCA1*	c.68_69del	p.Glu23Valfs*17	35377490	Pakistani	3800
*BRCA1*	c.685del	p.Ser229Leufs*5	35377490	Pakistani	3800
*BRCA1*	c.1471C > T	p.Gln491*	35377490	Pakistani	3800
*BRCA1*	c.1793 T > G	p.Leu598*	35377490	Pakistani	3800
*BRCA1*	c.2269del	p.Val757Phefs*8	35377490	Pakistani	3800
*BRCA1*	c.2340_2343del	p.Glu781Valfs*10	35377490	Pakistani	3800
*BRCA1*	c.2405_2406del	p.Val802Glufs*7	35377490	Pakistani	3800
*BRCA1*	c.2603C > G	p.Ser868*	35377490	Pakistani	3800
*BRCA1*	c.3339_3341del	p.Tyr1113*	35377490	Pakistani	3800
*BRCA1*	c.3598C > T	p.Gln1200*	35377490	Pakistani	3800
*BRCA1*	c.3770_3771del	p.Glu1257Glyfs*9	35377490	Pakistani	3800
*BRCA1*	c.4065_4068del	p.Asn1355Lysfs*10	35377490	Pakistani	3800
*BRCA1*	c.4183C > T	p.Gln1395*	35377490	Pakistani	3800
*BRCA1*	c.4485-1G > A	–	35377490	Pakistani	3800
*BRCA1*	c.4508C > A	p.Ser1503*	35377490	Pakistani	3800
*BRCA1*	c.5035del	p.Leu1679*	35377490	Pakistani	3800
*BRCA1*	c.5074 + 1G > A	–	35377490	Pakistani	3800
*BRCA1*	c.5278-1G > C	–	35377490	Pakistani	3800
*BRCA1*	c.5361_5362del	p.Cys1787Trpfs*42	35377490	Pakistani	3800
*BRCA1*	c.5503C > T	p.Arg1835*	35377490	Pakistani	3800
*BRCA1*	c.3228_3229del	p.Gly1077AlafsTer8	18821011	Italian	3225
*BRCA1*	c.3331_3334del	p.Gln1111AsnfsTer5	33087180	Iberia	2400-1600
*BRCA1*	c.4327C > T	p.Arg1443Ter	15883839	Canadian	2000
*BRCA1*	c.5266dup	p.Gln1756ProfsTer74	21119707	European	1800
*BRCA1*	c.676del	p.Cys226ValfsTer8	26852130	Northeastern Italian	1720
*BRCA1*	c.4035del	p.Glu1346LysfsTer20	23274591	Baltic	1550
*BRCA1*	c.3048_3052dup	p.Asn1018MetfsTer8	11781691	Western Swedish	1500
*BRCA1*	c.68_69del	p.Glu23ValfsTer17	26595274	Ashkenazi Jewish	1500–750
*BRCA1*	c.548-?_4185 +? del ex9-12del	–	25716084	Mexican	1440
*BRCA1*	c.815_824dup	p.Thr276Alafs	32025337	Senegal	1400
*BRCA1*	c.3626del	p.Lys1208_Leu1209insTer	11039575	Finnish	720–460
*BRCA1*	c.5309G > T	p.Gly1770Val	35216584	North African	800
*BRCA1*	c.1380dup	p.Phe461IlefsTer19	18215206	Italian	750
*BRCA1*	c.1016dup	p.Val340GlyfsTer6	16509964	Norse, Dutch, Italian	600
*BRCA1*	c.1556del	p.Lys519ArgfsTer13	11720839	Norwegian	600
*BRCA1*	c.697_698del	p.Val233AsnfsTer4	11720840	Norwegian	500
*BRCA1*	c.2641G > T	p.Glu881Ter	15146556	South African	500
*BRCA1*	c.5153-1G > A	p.?	19912264	Spanish	380
*BRCA1*	c.5212G > A	p.Gly1738Arg	17902052	Greek	275
*BRCA1*	c.4096 + 3A > G	p.?	11039576	Finnish	200
*BRCA1*	c.2685_2686del	p.Pro897LysfsTer5	15010701	Dutch	200
*BRCA1*	c.4097-2A > G	–	11039575	Finnish	< 200
*BRCA1*	c.1175_1214del	p.Leu392GlnfsTer5	8571953	Unknown	180
*BRCA2*	c.9026_9030del	p.Tyr3009SerfsTer7	12655574	Northeast Spanish	2760
*BRCA2*	c.156_157insAlu	–	34087993	Portuguese	2600-2400
*BRCA2*	c.5116_5119del	p.Asn1706LeufsTer5	19949853	Spanish	1904
*BRCA2*	c.3036_3039del	p.Ser1013IlefsTer29	9585613	North American	1600
*BRCA2*	c.9310_9311del	p.Lys3104ValfsTer6	19949853	Spanish	1365
*BRCA2*	c.5146_5149del	p.Tyr1716LysfsTer8	19949853	Spanish	1200
*BRCA2*	c.5946del	p.Ser1982ArgfsTer22	9585613	Ashkenazi Jewish	580
*BRCA2*	c.771_775del	p.Asn257LysfsTer17	8673089	Icelanders	500
*BRCA2*	c.7480C > T	p.Arg2494Ter	11039581	Finnish	400–200
*BRCA2*	c.755_758del	p.Asp252ValfsTer24	9585613	North American/France	360
*BRCA2*	c.5771_5774del	p.Ile1924fs	33643918	Southern African	> 250
*BRCA2*	c.7934del	p.Arg2645Asnfs	33643918	Southern African	> 250
*BRCA2*	c.9118-2A > G	p.?	11039580	Finnish	220–140
*BRCA2*	c.8327 T > G	p.Leu2776Ter	11039580	Finnish	220–140
*MLH1*	c.306 + 5G > A	–	20858721	Spanish	2200
*MLH1*	c.1865 T > A	p.Leu622His	20858721	Spanish	425
*MSH2*	c.1457_1460del	p.Asn486fs	15042510	Southern Chinese	1560
*MSH2*	c.-823_1076 + 5984del	–	14871915	North American	313
*MSH2*	c.2152C > T	p.Gln718Ter	30968502	Portuguese	273
*MSH6*	c.10C > T	p.Gln4Ter	25318681	French Canadian	543
*MUTYH*	c.1103G > A	p.Gly368Asp	23361220	European	8675
*MUTYH*	c.452A > G	p.Tyr151Cys	23361220	European	7500
*MUTYH*	c.849 + 3A > C	–	22865608	Italian	2075
*TP53*	c.1010G > A	p.Arg337His	26618902	Brazilian	2000

### Evolution selection and human DDR PVs

**Fig. 5:**
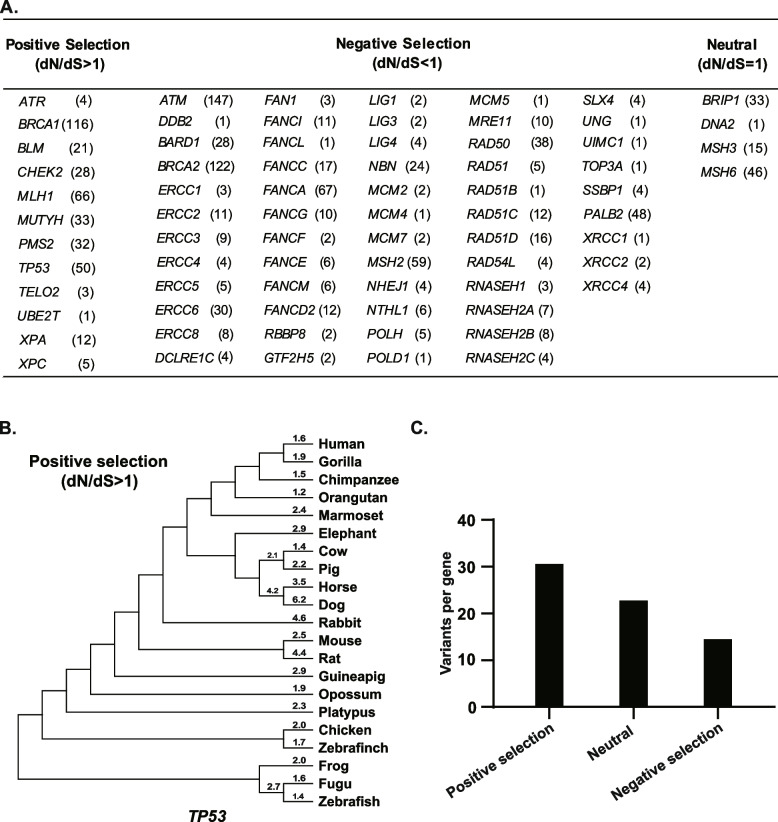
Relationship between evolution selection and DDR genes. It shows the type of evolution selection in the 73 PV-containing DDR genes. The PAML program was used for the test in 21 vertebrate species. **A** Summary of the DDR genes under positive, negative, and neutral selection. It shows that most of DDR genes were under negative selection. The number in parentheses refers to the PVs identified in ancient humans as list in Table 4. **B** *TP53* under positive selection. It shows that *TP53* is under positive selection in all 21 species tested but with different dN/dS ratios in different species. **C** Number of PV per DDR gene in each type of selection group. It shows that the positive selection group had the highest number, the neutral group had the intermediate number, and the negative group had the lowest number of PVs per gene

To know if the DDR PVs shared between modern and ancient humans were related with different type of evolution selection, we used the PAML program to calculate the dN/dS ratio for each DDR gene [37] and to determine their selection type in 21 species by using the positive-selected *BRCA1* as the control [51, 52]. The results showed that of the 73 DDR genes, 12 DDR genes were under positive selection, 4 DDR genes were neutral, and 57 DDR genes were under negative selection (Fig. 5A). For example, *BRCA1, TP53,* and *MLH1* were under positive selection within Primate (human, chimpanzee, gorilla, orangutan, marmoset) (Fig. 5B). Fig. S1 showed the actual *TP53* sequence alignment among the 21 species; *MUTYH*, *CHEK2*, *PMS2* and *UBE2T* were under positive selection between human and other 20 vertebrate species. Consistent with their suppression by negative selection, the PV rate of the 57 DDR genes in the total PVs of 73 DDR genes was low with only 14 PVs per gene, comparing to 30.9 PVs per gene in the positive selection group and 23.7 per gene in the neutral group (Fig. 5C). Besides the selection types, individual genes can also have their unique features. For example, while *BRCA1* was under positive selection and *BRCA2* was under negative selection, both were rich in PVs sharing between ancient and modern humans: *BRCA1* had 116 shared PVs and *BRCA2* had 122 shared PVs. We also compared the relationship between coding length of DDR genes and number of PVs for the 73 DDR genes with PVs shared between modern and ancient humans. The results showed that there was little correlation, implying that the physical coding length is not a determining factor for the shared DDR PVs (Supplementary Fig. 2, Supplementary Table 6).

## Discussion

Through systematical evolutionary analysis, our study reveals that nearly all DDR PVs in modern humans were not inherited by cross-species conservation, but arose within recent human evolution history, mostly within the past 5000 years. The results can be attributed by the great expansion of human population after the last glacial period of 10,000 years BP [53] and the agricultural revolution afterwards [54], as it increased the probability of generating new DDR PVs at the fixed rate of genetic variation. The results are consistent with the observation that genetic variants in modern humans were mostly arisen in recent human history [55, 56], and are also supported by the short history of nearly all DDR founder PVs identified in modern humans [39].

The logic of performing phylogenetic study to search for human DDR PVs in non-human vertebrates is that evolution conservation is widely used in human genetic variation analysis [57], although there have been no systematic evidence to prove or disprove whether human DDR PVs were evolutionarily conserved across different species (Supplementary Table 1). The results from our phylogenetic study clearly demonstrate that human DDR PVs were not inherited from non-human species. The result has a direct impact on the use of evolution conservation to annotate human DDR PVs: there is no biological basis for using evolution conservation to annotate human DDR PVs as they were not originated from cross-species conservation. The presence of human DDR PVs in evolutionarily distal species may simply reflect the coincidence of the same coding variants occurring in different species without biological significance [20]. The same could be appliable for the PVs in other human genes.

The frequency of the DDR PVs shared between modern and ancient humans is a critical issue for understanding the impact of evolution selection on DDR PVs. The comparison between the 1781 DDR PVs identified in modern humans and the 1266 DDR PVs in ancient humans showed that DDR PVs between ancient and modern humans shared similar patterns for most of the DDR PVs as exemplified by these at higher frequency in *BRCA2*, *ATM*, *BRCA1*, intermediate frequency in *MUTYH* and lower frequency in *XRCC1*. The data highlights that the abundance of DDR PVs in modern humans were already formed in the ancient humans (Fig. 4). Consistent with the negative selection for most of the DDR genes, the PVs in negatively selected DDR genes were at lower frequency in both modern and ancient humans. The PVs in *TP53*, *MLH1* and *MSH2* were exceptionally at higher frequency in ancient humans than in modern humans, suggesting that the PVs in these genes were suppressed to decrease their presence in modern humans due to their deleterious effects.

Evolution selection can play important roles in DDR PVs in ancient and modern humans. Positive selection can lead to higher number of PVs in the positively selected DDR genes (Fig. 5A). For example, more than 35,000 variants have been identified so far in human *BRCA1* (https://brcaexchange.org/factsheet, accessed June 6, 2023), as *BRCA1* is under strong positive selection [51, 58]. Furthermore, the relatively shorter time of PVs arisen may not allow evolution selection to function effectively as evidenced that nearly all *BRCA1* PVs carriers identified in modern humans are heterozygotic [59]. Other possibility could be that certain high-prevalent PVs in human population could be beneficial to be selected. For example, besides its classical DNA damage repair function, BRCA1 gains multiple new functions including regulation of immunity against viral infection [52], gene expression [60], neural development [61], and reproduction [62]. This may explain why the PVs in many DDR genes are persistently present in ancient and modern humans despite their obvious oncogenic effects. For example, *BRCA* PVs are highly prevalent of 0.2–0.5%, or one carrier in a few hundreds of individuals in modern humans [63–67]. The negatively selected genes had the lower number of PVs as expected due to their deleteriousness (Fig. 5C). *ATM*, *BRCA1* and *BRCA2* had the highest number of PVs among all the DDR genes. However, *BRCA1* is under positive selection whereas *ATM* and *BRCA2* are under negative selection. Even under negative selection, the extreme large size of *ATM* and *BRCA2* (*ATM*: 3056 amino acid residues, *BRCA2* 3418 amino acid residues) may still contribute to their high PVs numbers as their large sizes could provide higher probability of PV occurrence than these genes with smaller size under the same mutation rate.

It is well recognized that modern humans inherited genetic materials from extinct Neanderthals by admixture [68, 69]. The identification of multiple DDR PVs shared between Neanderthals and modern humans is consistent with the observation, and provided further evidence that Neanderthals may also contribute to cancer susceptibility in modern humans.

There are limitations in our study. The inclusion of 169 DDR genes in our analysis may not cover the whole set of genes involved in DDR activity, as different definition can be used to define DDR genes resulting in different number of DDR genes [70]. The use of the pathogenic variants from ClinVar may not cover the pathogenic variants from other resources and some PVs classified by ClinVar could be non-pathogenic, although ClinVar is considered as a high-quality standard in genetic variation classification and is widely used as the reference for clinical applications. It is also necessary to indicate that the firstly identified PV in a DDR gene may not be the earliest PV in ancient humans, as the same PV could occur in the ancient humans older than the first carrier identified in our study, although the possibility should not be high as the ancient non-PV samples older than the carriers of firstly identified PV were present in all PVs (Fig. 3, Supplementary Table 7). Another issue is for the reliability of the PVs identified in ancient humans considering the fact that many genomic sequences from ancient humans are often in poor quality. The PVs in ancient humans were identified by not only directly calling in ancient human but also by referring to the DDR PVs present in modern humans from the ClinVar database, which is based on rich supporting evidence, critical quality control, and expert evaluation. The combined evidences should enhance the reliability of the PVs identified in ancient humans. For the same PVs shared between ancient humans and modern humans, it is important to consider a possibility that certain shared PVs could occur independently between ancient humans and modern humans instead of transmitted genetically from ancient humans to modern humans. This was evidenced by certain *TP53* hotspot PVs at the loci with high spontaneous mutation that the same PV can occur spontaneously at the same location after their elimination [21, 71, 72]. With sufficiently large sample sizes, detecting PVs occurred independently in both modern and ancient humans could be plausible. However, the small size of ancient humans and large size of modern humans limits the significance of such comparison.

## Conclusions

Based on the data from our study, we conclude that human DDR PVs mostly arose in recent human history. Based on the data from our study, we propose a model to explain the evolutionary origin of DDR PVs in modern humans: the early human immigrants from Africa maintained low population size with limited DDR PVs inherited from their African ancestors. The expansion of human population in the last 10,000 years and evolution selection led to the substantial arising of human DDR PVs in modern humans. The bipartite effects of benefits and deleteriousness of certain DDR PVs can enhance the survival and reproduction of the human population but at the cost of increased cancer risk in the post-reproductive stage for the DDR PV carriers in human population.

### Supplementary Information

**Supplementary Material 1.**

**Supplementary Material 2.**

**Supplementary Material 3.**

**Supplementary Material 4.**

**Supplementary Material 5.**

**Supplementary Material 6.**

**Supplementary Material 7.**

**Supplementary Material 8.**

**Supplementary Material 9.**

## Data Availability

Only publicly available data were used in this study. The procedure of data analysis was described in the Materials and Methods. The data from the study are provided as supplementary tables and the online database dbDDR-Ancient humans, https://genemutation.fhs.um.edu.mo/dbDDR-AncientHumans. Further information is available from the corresponding author upon requests.

## Abbreviations

aDNA: Ancient DNA
BP: Before present
BV: Benign Variant
DDR: DNA Damage Repair
DR: Direct Reversal
HPCC: High-Performance Computing Cluster
HR: Homologous Recombination
MEGA: Molecular Evolutionary Genetics Analysis
MMR: Mismatch Repair
MYA: Million Years Ago
PARPi: PARP inhibitors
PV: Pathogenic Variant
SNV: Single Nucleotide Variant
