# Supplementary material for: Pathogenic variants in human DNA damage repair genes mostly arose in recent human history
Source: BMC Cancer. 2024 Apr 4;24:415. doi: 10.1186/s12885-024-12160-6 (PMC10993466; doi:10.1186/s12885-024-12160-6)
Supplement: Supplementary file 1 — Supplementary Material 1. [file 12885_2024_12160_MOESM1_ESM.pdf]

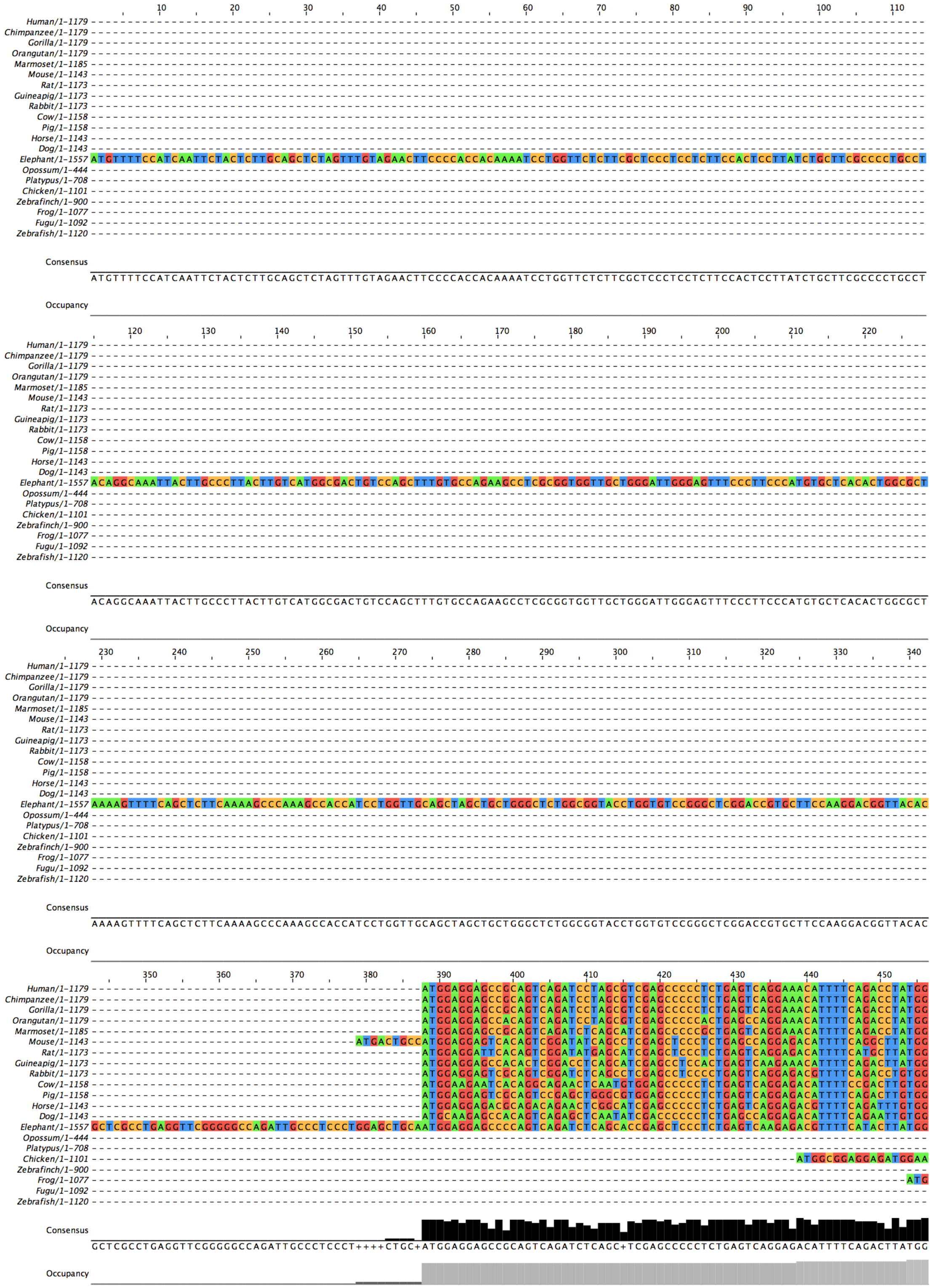

Human/1-1179
Chimpanzee/1-1179
Gorilla/1-1179
Orangutan/1-1179
Marmoset/1-1185
Mouse/1-1143
Rat/1-1173
Guineapig/1-1173
Rabbit/1-1173
Cow/1-1158
Pig/1-1158
Horse/1-1143
Dog/1-1143
Elephant/1-1557
Opossum/1-444
Platypus/1-708
Chicken/1-1101
Zebrafinch/1-900
Frog/1-1077
Fugu/1-1092
Zebrafish/1-1120
10 20 30 40 50 60 70 80 90 100 110
Consensus
ATGTTTTCCATCAATTCTACTCTTGCAGCTCTAGTTTGTAGAACTTCCCCACCACAAAATCCTGGTTCTCTTCGCTCCCTCCTCTTCCACTCCTTATCTGCTTCGCCCCTGCCT
Occupancy
120 130 140 150 160 170 180 190 200 210 220
Consensus
ACAGGCAAATTACTTGCCCTTACTTGTCATGGCGACTGTCCAGCTTTGTGCCAGAAGCCTCGCGGTGGTTGCTGGGATTGGGAGTTTCCCTTCCCATGTGCTCACACTGGCGCT
Occupancy
230 240 250 260 270 280 290 300 310 320 330 340
Consensus
AAAAGTTTTCAGCTCTTCAAAAGCCCAAAGCCACCATCCTGGTTGCAGCTAGCTGCTGGGCTCTGGCGGTACCTGGTGTCCGGGCTCGGACCGTGCTTCCAAGGACGGTTACAC
Occupancy
350 360 370 380 390 400 410 420 430 440 450
Consensus
GCTCGCCTGAGGTTCGGGGGCCAGATTGCCCTCCCT++++CTGC+ATGGAGGAGCCGCAGTCAGATCTCAGC+TCGAGCCCCCTCTGAGTCAGGAGACATTTTCAGACTTATGG
Occupancy

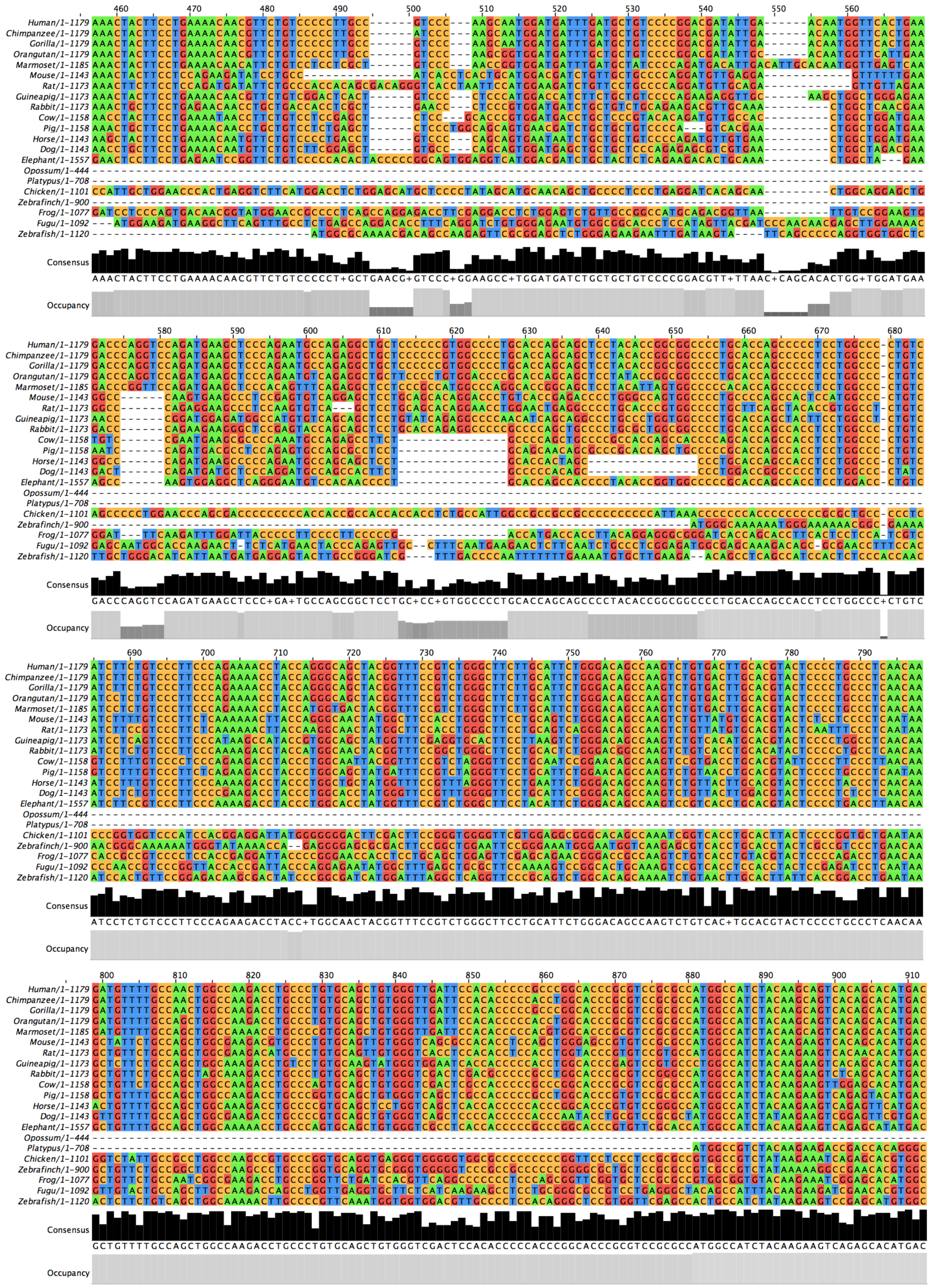
Human/1-1179
Chimpanzee/1-1179
Gorilla/1-1179
Orangutan/1-1179
Marmoset/1-1185
Mouse/1-1143
Rat/1-1173
Guineapig/1-1173
Rabbit/1-1173
Cow/1-1158
Pig/1-1158
Horse/1-1143
Dog/1-1143
Elephant/1-1557
Opossum/1-444
Platypus/1-708
Chicken/1-1101
Zebrafinch/1-900
Frog/1-1077
Fugu/1-1092
Zebrafish/1-1120
Consensus
Occupancy

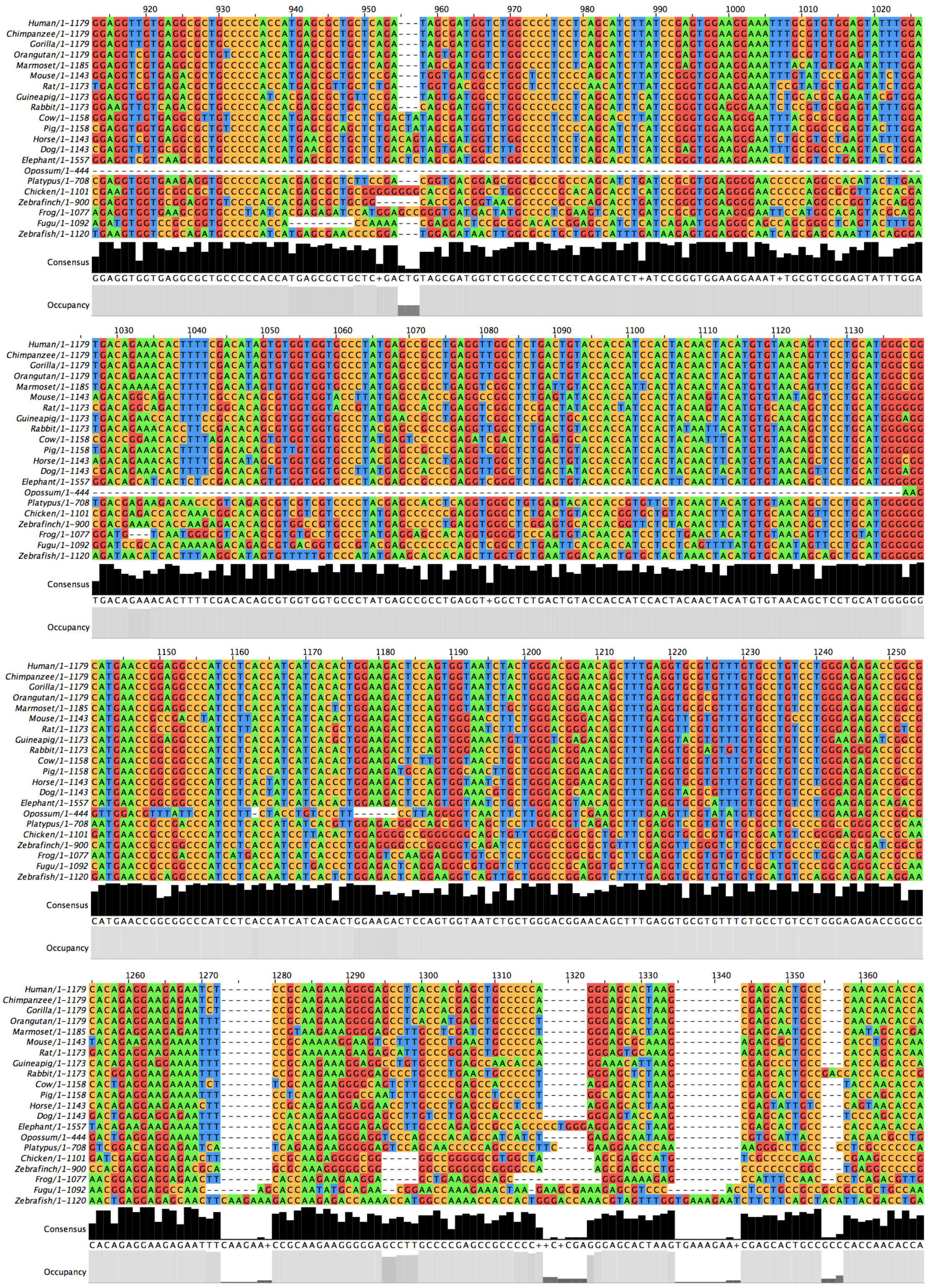
Human/1-1179
Chimpanzee/1-1179
Gorilla/1-1179
Orangutan/1-1179
Marmoset/1-1185
Mouse/1-1143
Rat/1-1173
Guineapig/1-1173
Rabbit/1-1173
Cow/1-1158
Pig/1-1158
Horse/1-1143
Dog/1-1143
Elephant/1-1557
Opossum/1-444
Platypus/1-708
Chicken/1-1101
Zebrafinch/1-900
Frog/1-1077
Fugu/1-1092
Zebrafish/1-1120
Consensus
Occupancy

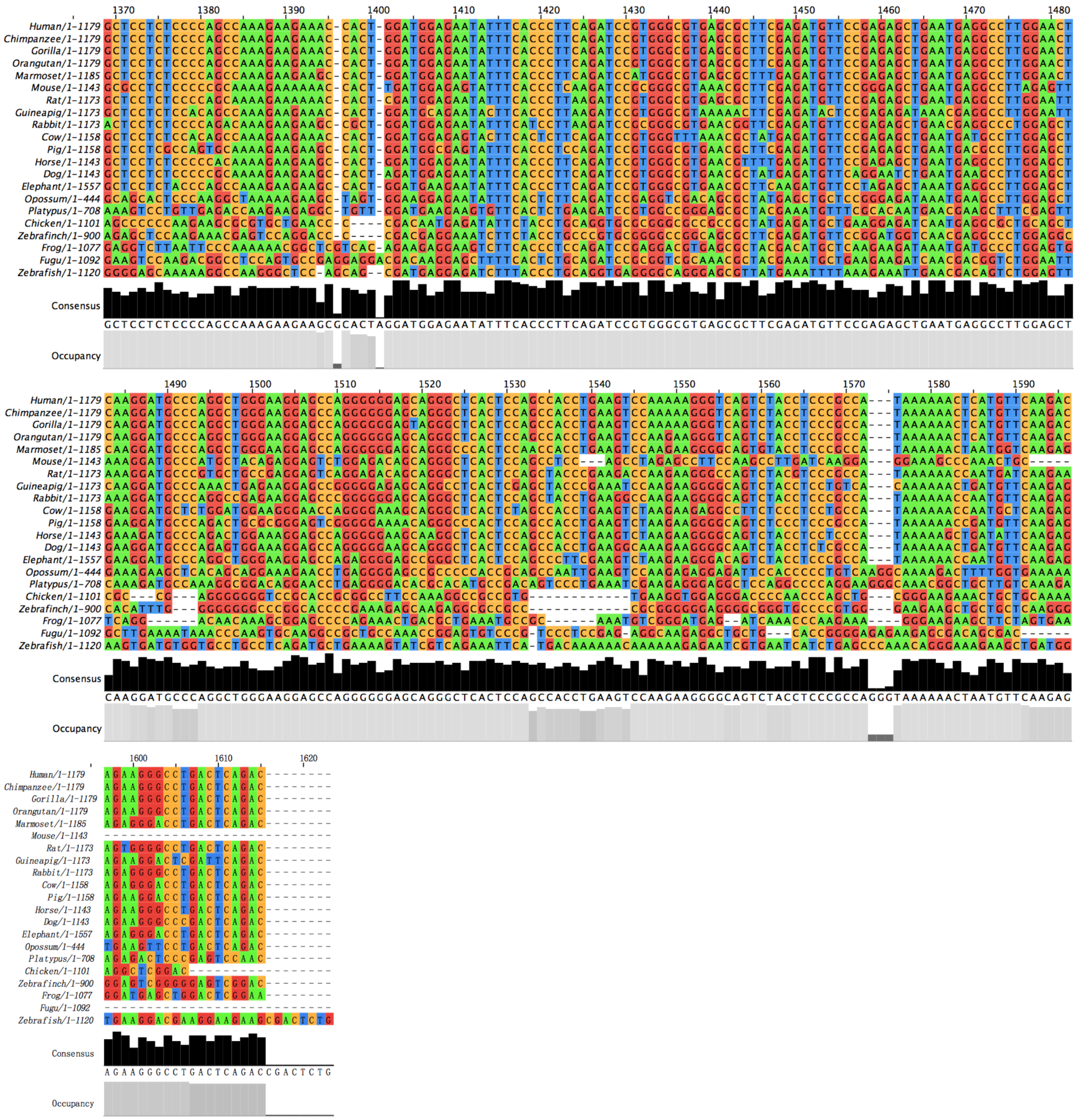

Figure S1. Multiple sequence alignment of *TP53* cDNA for 21 species. Sequence alignment is shown in color per nucleotide. Green: A, Orange: C, Red: G, Blue: T. The consensus bar chart represents the nucleotide bases. “-”: shared among species.
