# Supplementary material for: Pathogenic variants in human DNA damage repair genes mostly arose in recent human history
Source: BMC Cancer. 2024 Apr 4;24:415. doi: 10.1186/s12885-024-12160-6 (PMC10993466; doi:10.1186/s12885-024-12160-6)
Supplement: Supplementary file 2 — Supplementary Material 2. [file 12885_2024_12160_MOESM2_ESM.pdf]

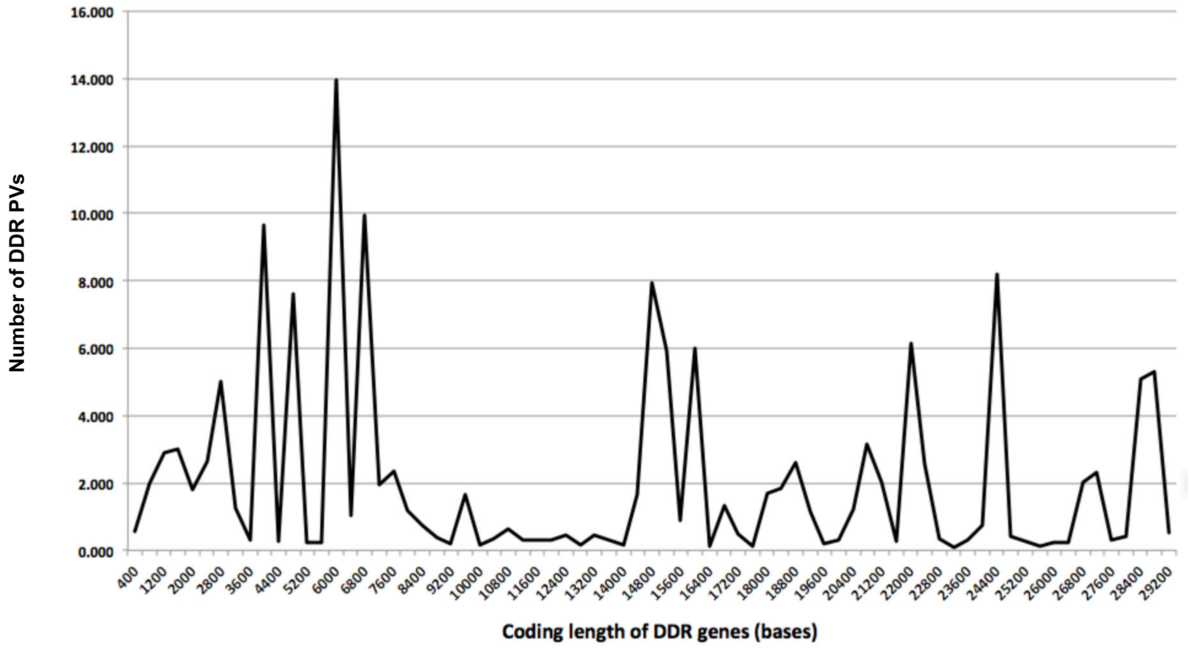

Figure S2. Relationship between coding length of DDR genes and number of PVs. The coding length for each of the 73 DDR genes with PVs shared between modern humans and ancient humans were plotted with the number of PVs in each of the 73 genes. The results show that the number of PVs didn't increase following the increased length, indicating that there is little correlation between the coding length and the number of shared PVs in the 73 DDR genes.
